# Bleeding Meckel's Diverticulum in Children: The Diagnostic Value of Double-Balloon Enteroscopy

**DOI:** 10.1155/2017/7940851

**Published:** 2017-03-22

**Authors:** Lan-Lan Geng, Pei-Yu Chen, Qiang Wu, Hui-Wen Li, Ding-You Li, Min Yang, Si-Tang Gong

**Affiliations:** ^1^Jinan University, Guangzhou, Guangdong, China; ^2^Department of Gastroenterology, Guangzhou Women and Children's Medical Center of Guangzhou Medical University, Guangzhou, Guangdong, China; ^3^Division of Gastroenterology, Children's Mercy Hospital, Kansas City, MO, USA

## Abstract

*Background*. Meckel's diverticulum (MD) is the most common congenital anomaly of the gastrointestinal tract. The purpose of this study was to evaluate the diagnostic value and safety of double-balloon enteroscopy (DBE) for bleeding MD in children. *Methods*. We included consecutive children who were highly suspected of MD between 2012 and 2013. All patients underwent Meckel's scan. DBE was performed for patient with negative Meckel's scan. An exploratory laparoscopy was performed in children with positive Meckel's scan or DBE. *Results*. 42 patients met the inclusion criteria. 40 patients were confirmed to have MD by exploratory laparoscopy. Meckel's scan was positive in 36 and negative in 6, with 34 as true positives and 2 as false positives. Six patients with negative Meckel's scan were found to have MD by retrograde DBE and had immediate operation. The distance from the diverticulum to the ileocecal valve was 40 to 60 cm. Ectopic gastric mucosa was present in all 6 patients (100%). After operation, patients were followed in clinic for 20 to 42 months and no evidence of GI bleeding or recurrent anemia was observed. *Conclusions*. Double-balloon enteroscopy can be a reliable diagnostic tool for bleeding Meckel's diverticulum in children with negative Meckel's scan.

## 1. Introduction

Meckel's diverticulum (MD) is the most common congenital anomaly of the gastrointestinal tract, occurring in approximately 1% to 3% of the population with a male-to-female ratio ranging from 1.7 : 1 to 2.4 : 1 [1, 2]. This congenital anomaly becomes clinically apparent only in patients with complications (e.g., GI tract hemorrhage, intestinal obstruction, or diverticulitis) which are reported to occur in 19% of cases [2]. GI tract hemorrhage is reported as the most common complication of a MD in the pediatric population [3]. Bleeding may manifest clinically as acute massive hemorrhage, anemia due to chronic bleeding, or self-limiting recurrent episodes. Hemorrhage is thought to be due to peptic ulcerations in the ectopic gastric mucosa or rarely from adjacent ileal ulcers secondary to acid secreted in a MD.

The diagnosis of bleeding MD is challenging. Technetium-99m pertechnetate scan (Meckel's scan) is currently the standard noninvasive method for preoperative diagnosis of MD in children with lower GI hemorrhage, with a sensitivity of 85% and specificity of 95% [4]. A repeat Meckel's scan is recommended if the initial scan is negative or equivocal, but clinical suspicion for MD is still high or patient preparation is inappropriate [5].

Other noninvasive diagnostic methods, including radiography, arteriography, ultrasonography, and computed tomography, have poor diagnostic value [6]. In addition, diagnostic laparoscopy or laparotomy may be necessary if there is still suspicion of a small diverticulum which was missed by other diagnostic tests. It is invasive and may pose an unwanted risk for patients who eventually show no cause for GI bleeding. Under such circumstances, double-balloon enteroscopy (DBE) may be beneficial. The use of DBE for the detection of Meckel's diverticulum was first described by Gasbarrini et al. [7]. However, uncertainty remains about the diagnostic value and safety of DBE in children. We report here 6 children who were highly suspected of having MD whose noninvasive diagnostic methods were negative, and their diagnoses were eventually confirmed by DBE.

## 2. Methods

We retrospectively reviewed clinical charts in a tertiary children's hospital in Guangzhou, China. We included consecutive children who presented with dark-red stools and/or melena, were highly suspected to have MD after a series of examinations, and were admitted in the GI department between January 2012 and December 2013. The main diagnostic tests include Meckel's scan and DBE. Meckel's scan was repeated once or twice during recurrent GI bleeding episode, if clinical suspicion for MD was still high after thorough investigations including abdominal ultrasonography, upper endoscopy, colonoscopy, capsule endoscopy (CE), and/or CT scan. Since Meckel's scan is noninvasive and relatively inexpensive (1/6 cost of DBE) with close to 100% positive predictive value, DBE was performed only if Meckel's scan was negative. Children were transferred to the surgery department for exploratory laparoscopy if Meckel's scan or DBE was positive for MD. Results of operation and histology as well as other diagnostic methods were reviewed.

The DBE system (Fujinon; Fujinon Inc., Japan) consists of a high-resolution video enteroscope (EN-450P5/28) with a flexible over-tube (TS-12140). The enteroscope has a working length of 200 cm and an outer diameter of 9.8 mm. The flexible over-tube has a length of 145 cm and outer diameter of 12.2 mm. Retrograde DBE was performed by one experienced gastroenterologist in the GI department.

This study was approved by the Institutional Ethics Committee of the Guangzhou Women and Children's Medical Center. Written informed consent was given by the caregiver of children for their clinical records to be used in this study.

## 3. Results

A total of 42 patients (31 males; median age 5.7 years; range 0.6–13 years) met the inclusion criteria. All children underwent exploratory laparoscopy; 40 patients were confirmed to have MD.

Among the 42 patients, Meckel's scan was positive in 36, with 34 as true positives and 2 as false positives: one was found to have an intestinal duplication and the other one had no lesion identified during surgery but was eventually diagnosed with intestinal telangiectasia when DBE was subsequently performed. Six children with negative Meckel's scan were considered false negatives since both DBE and exploratory laparoscopy confirmed diagnosis of MD. The sensitivity of Meckel's scan was 85% (34/40). Three patients were positive on a repeat Meckel's scan and were proved to be true positives by operation. Six patients remained negative on a repeat Meckel's scan and subsequently underwent DBE.

**Figure 1:**
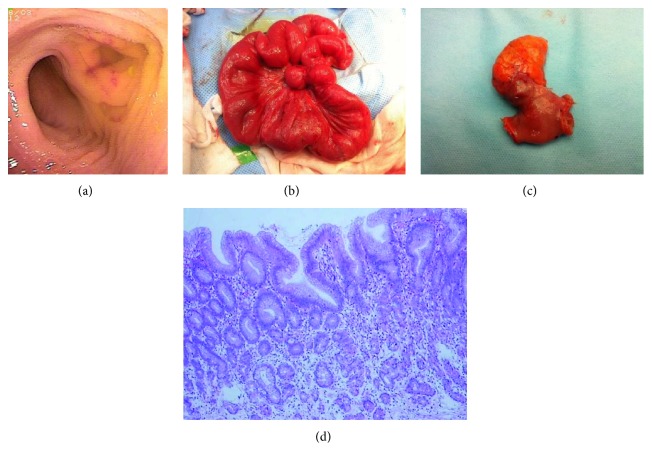
Double-balloon enteroscopy in a patient (case number 4). (a) Double lumen could be seen, and the right lumen was diverticulum. (b) Ileal diverticulum was seen on the other side of mesentery during operation. (c) Resected specimen of the patient. (d) Microscopic section shows Meckel's diverticulum demonstrating ectopic gastric mucosa (H&E, orig. mag. ×40).

Abdominal ultrasound detected MD in one patient who had positive Meckel's scan, and the diagnosis was confirmed by operation. Other diagnostic methods did not detect MD in any of the 42 patients. Six patients were diagnosed with MD by DBE and subsequently confirmed by operation (Figure 1). Their clinical characteristics, surgical methods, and features of MD are summarized in Tables 1 and 2. Among the 42 patients, 37 patients were initially presented to outside hospitals with no DBE available and had abdominal ultrasound and other diagnostic studies before they were transferred to our hospital.

As shown in Table 1, age of presentation was between 6 and 12 years, with male predominance. The course of disease was 4 days to 3 years, manifesting as recurrent and/or acute passage of dark-red stools and/or melena per rectum. Anemia was common with the lowest hemoglobin at 5.1–6.3 g/dl in 5 patients who required blood transfusion. Retrograde DBE examination found an ileal diverticulum in each subject. The typical change under DBE was a double lumen in one visual field in which one lumen was normal intestine and the other one a dead end; exploration of the dead end (diverticulum) demonstrated mucosal inflammatory changes with erythema, edema and/or erosion, and ulceration. After the diagnosis by DBE, immediate exploratory laparoscopy was performed in the 6 patients to avoid another anesthetic. Surgical methods were wedge diverticulectomy (4 patients) or end-to-end anastomosis after ileal resection (2 patients) due to the basal width of diverticulum. The distance from the diverticulum to the ileocecal valve was 40 to 60 cm. Diverticulum length ranged from 2.5 cm to 8.0 cm, and diameter ranged from 1.5 cm to 4.0 cm. Ectopic gastric mucosa was present in all 6 patients (100%). After DBE examination and operation, no complication occurred. Follow-up time was 20 to 42 months after surgery, and there was no evidence of GI bleeding or recurrent anemia in any of the 6 patients.

## 4. Discussion

MD is the most common cause for painless severe lower gastrointestinal bleeding in children and usually manifested as self-limiting recurrent and episodic rectal bleeding. Surgical resection is necessary once a diagnosis of MD is made. Accurate diagnosis prior to surgery is very important for patients. From January 2012 to December 2013, 42 children in our department were highly suspected of MD and transferred to surgical department for exploratory laparoscopy. MD was diagnosed in 40 children, which account for about 1/3 of the MD cases operated in the surgical department in this same period of time. Among the 42 children, Meckel's scan was positive in 36 patients, in whom 34 children were true positives, giving the sensitivity of Meckel's scan at 85%. Meckel's scan is the preferred method to detect bleeding MD [8, 9]. A repeat Meckel's scan is recommended if the initial scan is negative or equivocal but clinical suspicion for MD is still high. It was proved to be useful in 3 of our patients.

Six patients were negative on initial and/or repeat Meckel's scan but were subsequently proved to be false negatives by DBE examination and operation. Ectopic gastric mucosa was found in all 6 patients. The false negativity of Meckel's scan may be attributed to an insufficient ectopic gastric mucosa, diluted radioactivity due to hemorrhage or intestinal hypersecretion, or inadequate patient preparation such as premedication with pentagastrin, glucagon, and H^2^ blocker to increase sensitivity of Meckel's scan [5].

Nonspecific false-positive findings of Meckel's scan are not common, and potential causes include intestinal obstructive disease, intussusception, inflammatory disease, arteriovenous malformation, peptic ulcer, GI tumor, angioma, and anomaly of the urinary system (hydronephrosis, ectopic kidney, vesicoureteral reflex, and bladder diverticulum) [10]. Two children in our study were false positive for Meckel's scan and were subsequently diagnosed with intestinal duplication by operation and intestinal telangiectasia by a subsequent DBE examination following a negative surgical exploration, respectively.

The value of other diagnostic methods such as ultrasonography, upper endoscopy, colonoscopy, capsule endoscopy, and CT scan is very low in MD. In our study, ultrasonography was performed in 42 children but only positive in one child. Other examinations were performed in some children, but all of the results were negative. One promising method for MD detection is wireless capsule endoscopy. A recent study reported that capsule endoscopy detected 13 patients with MD and two were false positive, giving a positive predictive value of 84.6% [11]. In our study, three of the 6 patients who underwent DBE had prior negative capsule endoscopy. Further study would be needed before an extensive use of wireless capsule endoscopy for diagnosing MD.

Six patients in our study were diagnosed with MD by DBE examination and then confirmed by operation and pathology. We performed DBE in all 6 children by using retrograde transanal manner, and no complications occurred. It was reported that the mean distance from the diverticulum to the ileocecal valve was 34 cm in children aged < 2 years, 46 cm in patients aged 3 to 21 years, and 67 cm in adults aged 21 years or older [1]. This gives the rationale that retrograde transanal DBE can easily reach MD in children. Acute pancreatitis is a major concern and occurs in 0.3% of patients undergoing an antegrade DBE but is not an issue in patients undergoing retrograde DBE for detecting MD. Leung [12] suggested that retrograde DBE is suitable in children with a body weight of 31 lbs (14 kg) and above. In our patients, weights ranged from 22 to 49 kg.

There have been one case report and 2 case series reports of MD detected by DBE in Chinese children [13–15]. In addition, Fukushima et al. had reported 2 children with MD detected by DBE in Japan [16]. We further confirmed that DBE is an effective and safe diagnostic method and less invasive for patients who are suspected of MD but have negative noninvasive diagnostic procedures. DBE is only equipped in a few large medical centers in China due to lack of well-trained and experienced professionals, and therefore, its widespread use is still limited. When available, DBE should be considered the choice of diagnostic method for bleeding MD if Meckel's scan is negative.

## 5. Conclusions

Double-balloon enteroscopy can be a reliable diagnostic tool for bleeding Meckel's diverticulum in children with negative Meckel's scan.

## Figures and Tables

**Table 1:** Clinical characteristics of patients who underwent retrograde double-balloon enteroscopy for Meckel's diverticulum.

Case number	Age (y)/sex	Symptoms/course of disease	The lowest Hb (g/dl)	Prior examinations
1	7/M	Recurrent GI bleeding/3 years	6.0	Tc-99 scan, 3 times Abdominal US Upper endoscopy Colonoscopy GI barium meal Capsule endoscopy CT scan

2	10/F	Recurrent GI bleeding/3 months	5.6	Tc-99 scan, 1 time Abdominal US Upper endoscopy Colonoscopy CT scan

3	6/M	Recurrent GI bleeding/1 month	6.3	Tc-99 scan, 2 times Abdominal US Upper endoscopy Colonoscopy Capsule endoscopy CT scan

4	12/M	Recurrent GI bleeding/7 months	10.6	Tc-99 scan, 1 time Abdominal US Upper endoscopy Colonoscopy Capsule endoscopy CT scan

5	11/M	GI bleeding/4 days	5.1	Tc-99 scan, 2 times Abdominal US Upper endoscopy

6	8/M	Recurrent GI bleeding/1 year	5.8	Tc-99 scan, 1 time Abdominal US Upper endoscopy Colonoscopy CT scan

**Table 2:** Surgical method and features of patients who underwent retrograde double-balloon enteroscopy for Meckel's diverticulum.

Case number	Surgical method	Features of Meckel's diverticulum			
		Length (cm)	Diameter (cm)	Distance to ileocecal valve (cm)	Ectopic gastric tissue
1	Diverticulectomy	5	2.5	55	Yes
2	Diverticulectomy	4	2.8	60	Yes
3	Diverticulectomy	2.5	1.5	50	Yes
4	End-to-end anastomosis after ileal resection	3.5	2.0	40	Yes
5	End-to-end anastomosis after ileal resection	8	4	60	Yes
6	Diverticulectomy	3	2	60	Yes

## Additional Points

*Availability of Data and Material*. The data supporting the current findings are not publicly available since the database is currently not anonymous and contains all the patients' names. However, it will be available upon request.
